# Daily temperature profiles in and around Western Kenyan larval habitats of *Anopheles gambiae *as related to egg mortality

**DOI:** 10.1186/1475-2875-5-87

**Published:** 2006-10-12

**Authors:** Juan Huang, Edward D Walker, John Vulule, James R Miller

**Affiliations:** 1 Department of Entomology, Michigan State University, East Lansing, MI 48824, USA; 2 Microbiology and Molecular Genetics, Michigan State University, East Lansing, MI 48824, USA; 3 Kenya Medical Research Institute, Kisumu, Kenya

## Abstract

**Background:**

*Anopheles gambiae *eggs are more frequently found on soil around puddle habitats of the larvae, than on the water surface itself in Western Kenya. Thus, eggs can experience temperatures more wide-ranging and lethal than those experienced by larvae or pupae confined within puddles.

**Methods:**

Small batches of eggs from house-collected *An. gambiae *as well as from the Kisumu laboratory strain were bathed for defined times in water whose temperature was precisely controlled. Daily temperature profiles were recorded by an infrared thermometer on seven different days in and around three types of typical *An. gambiae *larval habitats at Kisian.

**Results:**

For wild eggs, significant mortality occurred upon brief heating between 42 – 44°C. Few eggs hatched after 10 min at 45°C and none hatched above this temperature. A similar pattern occurred for eggs of the Kisumu strain, except it was shifted downwards by 1°C. Egg mortality was time-dependent above 40°C. Temperatures of water in the three types of larval habitats never exceeded 35°C. Wet or damp mud rarely and only briefly exceeded 40°C; thus, water and mud would be highly conducive to egg survival and development. However, dry soils frequently reached 40 – 50°C for several h. Eggs stranded on dry surfaces would experience substantial mortality on hot, sunny days.

**Conclusion:**

Moist mud around puddles constitutes suitable habitat for *An. gambiae *eggs; however, eggs on the surface of dry soil under direct sunlight are unlikely to survive for more than a few hours.

## Background

Malaria infects 300–500 million people each year [1]. More than 90% of malarial cases occur in Sub-Saharan Africa, where *Anopheles gambiae *is the principal vector. Despite the high vectorial capacity of this mosquito, critical aspects of its biology bearing on population dynamics and ultimately malaria transmission remain to be elucidated. For example, little is known about how abiotic factors like temperature influence egg survival. Eggs of *An. gambiae *are more frequently found on mud (soil) around puddle habitats of the larvae than on the water surface itself [2]. Larvae developing and hatching on wet substrates like mud [3] can crawl to puddles [2,4] or perhaps be washed there by rains [2]. It is also not uncommon for puddles and the mud around them to desiccate. Thus, eggs can experience temperature regimes more wide-ranging and lethal than temperatures experienced by larvae or pupae in puddles.

Some research has been conducted on tolerance of *An. gambiae *eggs to desiccation. Unlike some mosquitoes, e.g., *Aedes *and *Ochlerotatus*, eggs of *An. gambiae *cannot tolerate prolonged desiccation [5]. Survivorship of *An. gambiae *eggs in drying soils held in the laboratory was found to be inversely related to time after deposition; very few eggs in drying soils hatched after 12 to 15 days upon re-flooded [6]. It has been suggested that the egg stage of *An. gambiae *might contribute to the short-term survival of this vector during dry periods [6]. However, these studies did not consider temperatures likely to be encountered when soils dry under natural, out-door conditions likely to be sunny.

The effects of temperature on embryonic development and egg hatching of *An. gambiae *have received little attention. In contrast, considerable data are available for other mosquitoes and insects generally [7-11]. Upper tolerable temperatures for eggs in these studies ranged from 33 to 48°C.

Growth, development, and survival of *An. gambiae *as influenced by constant temperatures between 10 and 40°C have been analyzed under laboratory conditions [12]. The optimal temperature for larval growth was 28°C, while maximal fitness of adults occurred between 28 and 32°C. Growth and development of instars 1–4 and pupae ceased at 40, 38, 36, 34, and 34°C, respectively, under constant temperature regimes [12]. However, the ability of *An. gambiae *eggs to withstand temperatures of 40°C and greater was not reported, nor was the effect of fluctuating temperatures evaluated.

The objectives of the current study were to: 1) establish lethal temperatures for *An. gambiae *eggs briefly exposed to elevated temperatures, and 2) determine whether and for how long eggs located in and around typical *An. gambiae *larval habitats would be exposed to damaging temperatures during the long rainy season in Kisumu, Kenya.

## Materials and methods

### Mosquitoes and bioassay conditions

Experiments were performed using eggs from two sources: feral females of the *An. gambiae *complex aspirated from houses near Kisian, Kenya between 15 April and 15 May 2005, and the Kisumu laboratory strain (*An. gambiae s. s*.) originating from the Kenyan Medical Research Institute (KEMRI) located near Kisumu. Eggs used were laid overnight and were 9–20 h old by the time of heat treatment. After oviposition, samples of randomly selected females were removed from each cage and placed into individual 1.5 mL centrifuge tubes, air-dried under silica for 3 days, and stored at 4°C for species identification within the *An. gambiae *complex by PCR using the methods of Scott et al. [13]. Seventy five individuals were tested.

### Egg survival in response to temperature and exposure times

Published heating methods for mosquito eggs have employed water baths [8], incubators [14], or specially designed devices [15]. Here, a thermal cycler, normally used for polymerase chain reaction, was used as a rapid and precise heating device. Between 50 to 100 randomly selected eggs from an ovipositional dish receiving several thousand eggs of a given *An. gambiae *population were carefully transferred by a fine brush into 40 μl of water in a 0.2 ml PCR tube (Dot Scientific Incorporated, Burton, MI, USA). Tubes were heated for 10 min in a Thermal Cycler (GeneAmp PCR System 9700, Applied Biosystems, Foster City, CA, USA) to 40, 41, 42, 43, 44, 45, 46, 47, or 48°C. Control batches of eggs were also transferred into PCR tubes, but not heated. These PCR tubes were kept at the room temperature (22°C) while the other groups of eggs were being heated. Treated and control eggs were transferred into 9-cm diameter Petri dishes with 30 ml water; exact numbers of eggs in each Petri dish were then counted. Numbers of emerged larvae were recorded every day at 25°C until eggs were 7 d old. Pilot tests had revealed no hatch for any treatments beyond 7 d. In each run of the experiment, three replicates were performed on each of the 10 total temperatures. The whole procedure was repeated another two times using different batches of eggs.

Eggs of the Kisumu laboratory strain were heated at 40, 41, 42, or 43°C for 10–160 min (depending on temperature) to record egg mortality as influenced by the exposure times indicated in Figure 3. Thereafter, eggs were handled as above. Each time point was replicated three times using a given batch of eggs. This procedure was then twice repeated using different batches of eggs.

### Daily temperature profiles in and around *Anopheles gambiae *larval habitats

**Figure 1:**
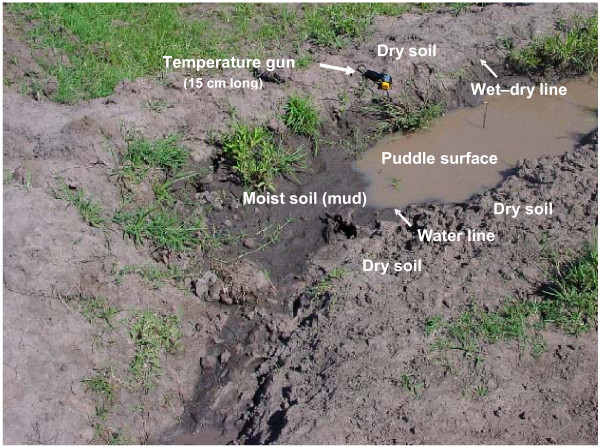
**Picture of an *Anopheles gambiae *larval habitat in a maize drainage ditch showing differentiation of zones where temperatures were monitored**. The shift from wet or damp mud to dry soil was abrupt and accompanied by a shift from black to gray.

Three typical types of *An. gambiae *larval habitats [16] were selected just outside the KEMRI campus in Kisian: puddle in a maize drainage ditch, puddle in a roadway, and a burrow pit. Daily temperature profiles in and around these larval habitats (Figure 1) were monitored once each h from 8:00 to 18:00 h by a hand-held infrared thermometer (Model IRT4, Spectrum Technologies, Inc., Plainfield, IL, USA) for 7 d between 27 April to 12 May, 2005, the long rainy season when *An. gambiae *population peaks. Since the burrow pit was surrounded by grasses, no un-shaded dry soil was available. However, temperatures of a rock (ca. 40 cm-diameter) at the edge of this burrow pit were included.

### Statistical analysis

Mean egg survival after exposure to the 10 constant temperatures was compared using 1-way analysis of variance (ANOVA) on data transformed by arcsine of the square root [17]. Mean separations were performed by Tukey's significant difference (HSD). The effect of exposure times on egg survival was analyzed by linear regression.

## Results

### Egg survival in response to temperature and exposure times

**Figure 2:**
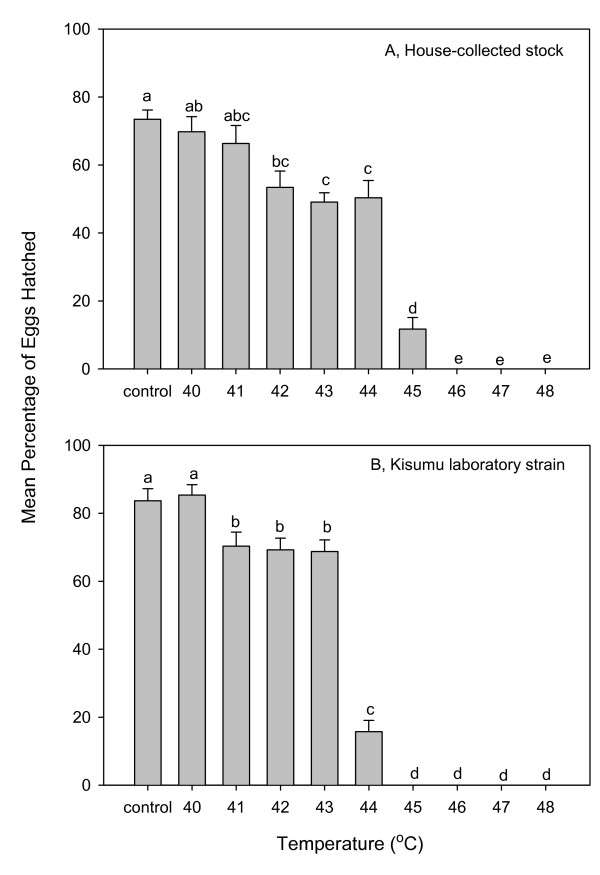
**Larval eclosion from two populations of *Anopheles gambiae *eggs as influenced by 10 min of exposure to elevated temperatures**. Error bars = S. E. M.

Of 75 house-collected mosquitoes identified by PCR, 71 were *An. gambiae s. s*., two were *Anopheles arabiensis*, and two were unknown. Therefore, eggs used in this study were overwhelmingly *An. gambiae s. s*. These eggs were tolerant of brief exposures of temperatures up to 44°C (Figure 2). Emergence of larvae after exposure to 42°C was significantly lower (F = 94; df = 9, 80; P < 0.001) than for control eggs from house-collected females. Eclosion dramatically decreased from over 50% to 12% with a temperature rise from 44 – 45°C. No eggs exposed to 46°C for 10 min hatched (Figure 2A). An identical pattern, but shifted 1°C lower, was recorded for eggs of the Kisumu laboratory strain (Figure 2B).

**Figure 3:**
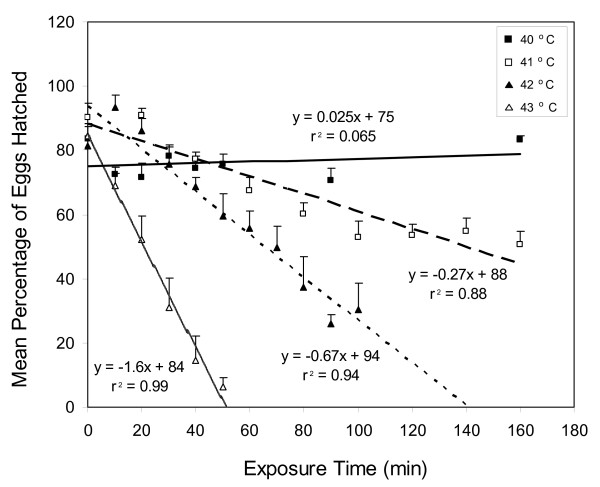
**The relationship between egg hatch and exposure times for *Anopheles gambiae *eggs heated at 40, 41, 42, or 43°C**. Error bars = S. E. M.

The survival of *An. gambiae *eggs at elevated temperatures was also influenced by exposure time (Figure 3). At given temperatures of 41, 42, and 43°C, the numbers of Kisumu eggs that hatched decreased linearly with exposure times (41°C: F = 49.0; df = 1, 7; P < 0.001; 42°C: F = 134.6; df = 1, 9; P < 0.001; 43°C: F = 396.8; df = 1, 4; P < 0.001). The lethal time for 50% kill (LT_50_) of eggs decreased from 126 to 20 min as exposure temperature increased from 41 to 43°C. However, no such pattern was observed at 40°C, where egg hatch was not significantly affected by exposure time up to 160 min (F = 0.4; df = 1, 6; P = 0.5).

**Figure 4:**
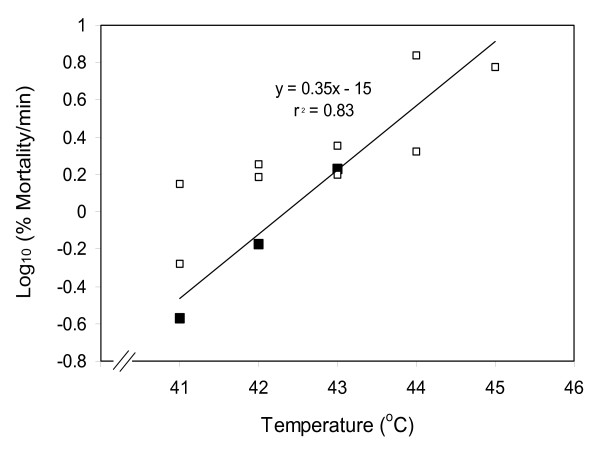
**Linear relationships between temperatures above 40°C and log_10 _% mortality/min for eggs of *Anopheles gambiae***. Data denoted by filled squares are the slopes from Figure 3; data denoted by open squares were calculated from Figure 2, based on mortality per 10 min for house-collected stock and Kisumu laboratory strain. % mortality/min for the open squares = (mean survivorship in control - mean survivorship in each treatment)/10 min. Because each slope from Figure 3 was a composite derived from 10 original data, these points (filled squares) were weighted 10× in a weighted regression analysis.

Egg survivorship appeared to diminish exponentially rather than linearly with increasing temperatures above 40°C. An exponential (logarithmic) relationship between temperature > 40°C and survivorship per minute of exposure at a given temperature was confirmed by Figure 4 (F = 183.7; df = 1, 37; P < 0.001). Moreover, these data yielded a general equation (log_10 _% mortality/min = 0.35(°C) - 15) permitting the calculation of egg mortality expected for various combinations of temperatures and times (Table 1).

**Table 1:** Predicted mortality of *Anopheles gambiae *eggs exposed to various combinations of temperature and time, as calculated from the regression equation of Figure 4.

	Predicted % Egg Mortality					
Time (min)	41°C	42°C	43°C	44°C	45°C	46°C

1	0.3	0.8	2	4	8	18
2	0.7	2	3	7	16	36
4	1	3	7	15	33	73
8	3	6	13	30	66	100
14	5	11	23	52	100	
20	7	15	33	74		
30	10	23	50	100		
60	20	45	100			
120	41	90				
180	61	100				
300	100					

### Daily temperature profiles in and around *Anopheles gambiae *larval habitats

**Figure 5:**
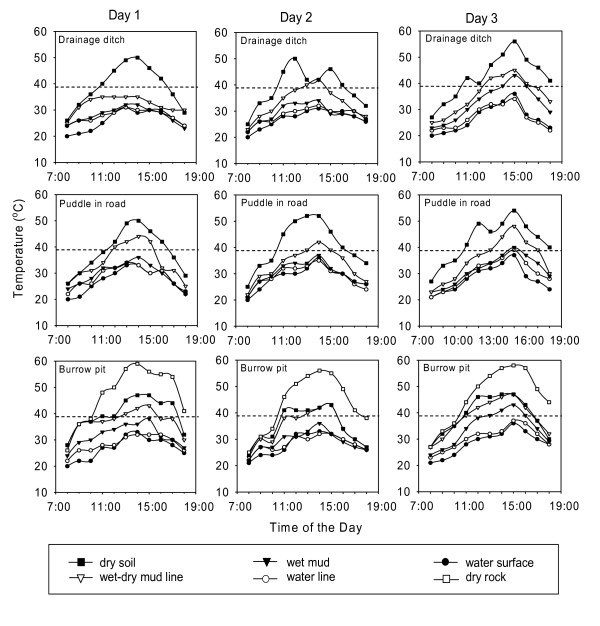
**Daily temperature profiles in and around three typical types of *Anopheles gambiae *larval habitats on three consecutive days (May 10, 11, and 12, 2005)**. The dotted line represents the upper tolerable temperature (40°C). Profiles recorded at similar sites on 4 other days were very similar. Table 1 reports outcomes across all the data.

Similar daily temperature profiles were recorded in and around the three types of larval habitats on each of the three consecutive sunny days shown in Figure 5. As expected, the maximum daily temperature occurred between 13:00–16:00 h. Soil temperatures rarely exceeded the upper tolerable temperature for *An. gambiae *eggs of 40°C, except where soils became dry to the touch and grey rather than black (Table 2). On sunny days, dry soils around habitats were very likely to exceed 41°C for an average of 4 h (Table 2). Temperatures at the water line and on wet or damp mud were little higher than the temperatures of puddle water.

**Table 2:** Frequency and duration of potentially lethal thermal events in and around *Anopheles gambiae *larval habitats between 27 April and 12 May, 2005 at Kisian, Kenya.

Location	Total events of 41°C or greater	Daily events per habitat	Mean duration per event (h ± S.D.)
Water surface	0	0	--
Water line	0	0	--
Wet or moist soil	2	0.1	1.5
Dry soil	15	0.7	3.9 ± 2

N = 21 habitat days; 15 habitat days were full sun.

## Discussion

It is well known that insect embryogenesis and egg hatching are influenced by temperature [10]. There are definite thresholds below or above which no eggs hatch. For example, all the embryos of *Culex theileri *died after eggs were incubated at a constant temperature between 39 to 42°C [11]. Exposure of eggs of *Culex quinquefasciatus *for 24 h at 39°C completely inhibited egg hatch [10]. All eggs of *Aedes structys *exposed to a constant 33°C failed to hatch [9]. No larvae emerged after *Anopheles sergentii *eggs were incubated at 34°C [18]. The upper tolerable temperatures for egg development and hatching of other insects were: 46 to 48°C for the tephritid fruit fly, *Bactrocera latifrons *[19]; 42°C for the Queensland fruit fly, *Bactrocera tryoni *[8]; 39°C for the common cattle grub, *Hypoderma lineatum *[20]; 37°C for the reindeer warble fly, *Hypoderma tarandi *[21]; and 32°C for the Diaprepes root weevil, *Diaprepes abbreviatus *[7].

Survival of *An. gambiae *eggs was strongly influenced both by temperature and exposure times (Figure 3; Table 1). The upper tolerable temperature for these eggs was 40°C. Above this threshold, the rate of egg kill was approximately linear over time for a given temperature (Figure 3). Estimated LT_50 _values for the following temperatures were: 41°C – 147 min; 42°C – 66 min; 43°C – 30 min; 44°C – 14 min; 45°C – 6 min; and 46°C – 2.8 min (based on the equation from Figure 4). Thus, for each successive degree temperature rose beyond 41°C, the time required for 50 % egg kill was approximately halved. Stated conversely, the velocity of some time-dependent process killing eggs approximately doubled with each rise of one degree C.

Protein denaturation [22] is a likely mechanism explaining this pattern of lethality. Nguyen et al. [24] quantified the time-course for denaturation of a firefly luciferase and an *Escherichia coli *β-galactosidase transfected into *Drosophila *and mouse cell lines. Denaturation was detectable at 37°C, but with a half-life of more than three h. Incubation of these cells at 42°C yielded approximately linear protein degradation profiles with half-lives ranging from 5–40 min depending upon experimental conditions. Mortality profiles in the current study using *An. gambiae *eggs strongly resemble these carefully quantified protein-denaturation profiles from *in vivo *and *in vitro *preparations using cell lines and proteins from organisms not known to be selected for high thermal tolerance. Such similarities suggest *An. gambiae *is not uniquely adapted to tolerate temperatures above those lethal to animal cells generally.

Ability to tolerate high temperatures can vary with life stage. Results from the current study combined with the findings of Bayoh and Lindsay [12] reveal that *An. gambiae *eggs are the most (40°C) and pupae and the 4^th ^instars the least (34°C) heat-tolerant life stages, respectively. Notably, the temperatures within our puddles sometimes exceeded the tolerance limits for larvae and pupae as measured under a constant-temperature rearing regimes [12]. Munga et al. [23] reported that the mean maximum daily water temperature in puddle habitats in open farmland was 38.8 ± 0.3°C. In the current study, that value was 31.3 ± 0.8°C (mean ± SEM). Although daily temperature profiles in and around these larval habitats were measured only for 7 days, data collected at a weather station [24] indicated that the mean air temperature was very stable throughout all of 2005. Further research is justified to document to what extent *Anopheles gambiae *adult production is limited by maximal temperatures within puddles.

Eggs from the laboratory strain were slightly more sensitive to heat stress than eggs from house-collected mosquitoes. Perhaps rearing for more than five years under no thermal stress caused the laboratory strain to lose a bit of thermal tolerance. Enhanced thermal tolerance of individual insects following non-lethal heat shock has been reported in some insects [8,25,26], including anopheline mosquito [26]. The effect of inducing heat-shock proteins in larvae of *Anopheles albimanus *was to increase the upper tolerable temperature by only 1.5°C. Thermal conditioning in *An. gambiae *eggs and interaction of temperature with humidity are worthy of additional investigation.

*An. gambiae *eggs appear to be thermally adapted only to residing on water or moist mud. Rapid evaporation of water from mud apparently has a pronounced cooling effect, making mud not much different thermally from water (Figure 5). Perhaps this is why *An. gambiae *readily oviposit on moist or wet soil [2]. However, sun-exposed dry soil is inhospitable to these eggs because it readily exceeds lethal temperatures. Eggs on the surface of dry soils baked for several hours under full sun will not survive and thus cannot contribute to *An. gambiae *populations and malaria transmission.

## Authors' contributions

JH and JRM designed and carried out the experiments, analysed and interpreted data, as well as drafted and revised the manuscript. EDW was P. I. of the grant supporting this work; he participated in study design, data interpretation, and revision of the manuscript. JV provided institutional support for this study. All authors read and approved the final manuscript.
